# Insomnia remission and improvement of bodily pain in older adults: a randomized clinical trial

**DOI:** 10.1097/PR9.0000000000001243

**Published:** 2025-02-05

**Authors:** Martin F. Bjurstrom, Richard Olmstead, Michael R. Irwin

**Affiliations:** a Department of Surgical Sciences, Uppsala University, Uppsala, Sweden; b Cousins Center for Psychoneuroimmunology, UCLA Semel Institute for Neuroscience, Los Angeles, CA, USA; c Department of Psychiatry and Biobehavioral Sciences, David Geffen School of Medicine, University of California, Los Angeles, CA, USA

**Keywords:** Insomnia, Aging, Bodily pain, CBT-I

## Abstract

**Introduction:**

Older adults with insomnia frequently report bothersome pain. Whether insomnia treatment reduces bodily pain in older adults without chronic pain conditions is not known.

**Objectives:**

This randomized controlled trial aimed to determine whether treatment of insomnia disorder with cognitive behavioral therapy for insomnia (CBT-I), as compared with sleep education therapy (SET), yields durable remission of insomnia and reduces moderate pain symptoms over 36 months in older adults with insomnia disorder.

**Methods:**

A community-based sample of 291 adults ages 60 years and older (mean age, 70.1 years; 57.7% female) with insomnia disorder, but no chronic pain condition, were randomized to 2 months of intervention with either CBT-I (n = 156) or SET (n = 135). The primary outcome was change in bodily pain as measured by the short form 36 (SF-36) health survey. Secondary outcome was remission of insomnia continuously sustained over 36 months of follow-up.

**Results:**

Improvements in bodily pain, as indexed by increases in SF-36 scores, were found in CBT-I with insomnia remission (adjusted β *=* 0.18; 95% CI, 0.004–0.360; *P* = 0.045) and in SET with insomnia remission (adjusted β = 0.25; 95% CI, 0.035–0.457; *P* = 0.023) but not in treatment groups without insomnia remission. As compared with those without insomnia remission, insomnia remission was associated with improvements in pain (adjusted β = 0.19; 95% CI, 0.047–0.325; *P* = 0.009) and with increases in the proportion of participants who achieved a minimal clinically important difference (likelihood ratio χ^2^_1,16_ = 264.04; *P <* 0.001).

**Conclusions:**

Sustained remission of insomnia disorder leads to improvements in bodily pain, with the potential to prevent chronic pain disorders in older adults with insomnia.

**Trial Registration:**

ClinicalTrials.gov NCT01641263.

## Abstract 2

Supplemental Digital Content is Available in the Text.

This randomized controlled trial found that sustained remission of insomnia reduced bodily pain over 36 months of follow-up in older adults with insomnia disorder but without a chronic pain condition.

## 1. Introduction

In the United States, more than half of community-dwelling older adults report bothersome pain,^30^ which is often undertreated in this population,^6,37^ leads to chronic nonmalignant pain conditions, and contributes to adverse physical and mental health outcomes.^8,9^ Effective strategies to address moderate pain symptoms are urgently needed, yet such efforts have been neglected for community-dwelling older adults, although older adults account for nearly 20% of the population in the United States, are most vulnerable to pain symptoms, and are at risk for chronic pain conditions.^32^

Insomnia also shows a similar high prevalence in older adults, with evidence that insomnia symptoms predict worsening of existing pain, spreading of pain, and onset of chronic pain conditions.^1,27,29^ Furthermore, in experimental studies, sleep loss induces hyperalgesia and increases self-reported pain.^13^ By contrast, less is known about the indirect benefits of insomnia treatment on self-reported pain. Among the nonpharmacologic treatments for insomnia, sleep education therapy (SET) is a behavioral program that targets behavioral factors that contribute to poor sleep. Another treatment, cognitive behavioral therapy for insomnia (CBT-I), which combines cognitive therapy, stimulus control, sleep restriction, sleep hygiene, and relaxation; CBT-I is recommended as the first-line treatment for insomnia disorder.^25^ Among adults who are comorbid for insomnia and chronic pain conditions, CBT-I is reported to reduce pain, although findings are limited by small sample sizes and short follow-up periods.^36^ However, to our knowledge, no research has evaluated whether CBT-I reduces pain symptoms in persons without chronic pain conditions. Furthermore, despite the high prevalence of pain symptoms in older adults, there is an absence of research in older adults with insomnia. A key uncertainty is whether behavioral treatments that target insomnia can effectively reduce moderate pain symptoms in older adults with insomnia disorder but without a chronic pain condition.

In this analysis of bodily pain, a secondary outcome of a selective depression prevention trial that targeted insomnia,^12^ we examine whether CBT-I vs SET will reduce bodily pain, as indexed by self-reported bodily pain score of the short form 36 (SF-36) health questionnaire (ie, SF-36 bodily pain) in community-dwelling older adults with insomnia disorder, but without a chronic pain condition. We hypothesized that insomnia remission, sustained over 36 months of follow-up, would account for linear changes in bodily pain.

## 2. Methods

### 2.1. Trial design and overview

This study is an analysis of an a priori secondary outcome, bodily pain, from an investigator-initiated, single site, parallel-group, randomized controlled, selective depression prevention trial.^12^ The trial protocol was previously described (Supplement, Protocol, http://links.lww.com/PR9/A282).^12^ Total enrollment was 291 participants, who provided written informed consent as approved by the UCLA (University of California, Los Angeles) Institutional Review Board. All data were deidentified. The trial data monitoring and steering committees of the UCLA Clinical Translational Sciences Institute oversaw the study, which was undertaken according to the intention-to-treat principle. This study adhered to the Consolidated Standards of Reporting Trials (CONSORT) reporting guideline.^38^

### 2.2. Participants

A community sample of adults ages 60 years or older was identified using a database of all available telephone numbers and mailing addresses of households with at least 1 person ages 60 years or older who resided within 15 miles of UCLA.^12^

Following screening eligibility for sleep disturbance (ie, Pittsburgh sleep quality index score >5)^7^ and absence of depression (ie, 10-item Center for Epidemiologic Studies–Depression score <4),^10^ diagnostic interviews confirmed (diagnostic and statistical manual of mental disorders, fourth edition) DSM-IV insomnia disorder and absence of DSM-IV or DSM-5 major depression or other psychiatric disorder within the past 12 months. Sleep disorders other than insomnia, such as sleep apnea, were excluded through interviews and questionnaires (eg, Berlin sleep apnea questionnaire).^28^ Complete exclusion criteria were previously described,^12^ including exclusion for chronic pain disorders requiring daily pain management and daily use of analgesics (eg, opioids). In the primary outcome trial,^12^ we excluded those with chronic pain conditions because pain disorders may have had independent, as well as indirect effects on insomnia and depression outcomes. Furthermore, analgesics may have effects on inflammation, which was examined as mediating pathway linking insomnia remission with the prevention of depression. Self-reported race and ethnicity satisfied National Institutes of Health requirements.

### 2.3. Procedures

As previously described,^12^ participants were randomly allocated in a 1:1 ratio to CBT-I or SET. Allocation concealment used sequentially numbered, opaque, sealed envelopes. Investigators and assessors were blind to allocation. Participants were blind to hypotheses and the content of the other treatment group through study duration.

Cognitive behavioral therapy for insomnia and SET interventions were delivered weekly, in the format of 120-minute group sessions over the course of 2 months. Cognitive behavioral therapy for insomnia is recommended as the first-line treatment for insomnia disorder^25,31,33^ and contains 5 components: cognitive therapy, stimulus control, sleep restriction, sleep hygiene, and relaxation. Sleep education therapy is a universal behavioral program that targets day-to-day behavioral and environmental factors that contribute to poor sleep,^24^ by providing education in sleep hygiene, sleep biology, characteristics of healthy and unhealthy sleep, and stress biology. As an active comparator condition, SET improves insomnia but is less effective and less durable than CBT-I.^16,24^

### 2.4. Outcomes

The outcome of this analysis was the bodily pain score of the SF-36 health questionnaire (ie, SF-36 bodily pain).^21,23^ Short form 36 bodily pain was an a priori defined secondary outcome from the selective depression prevention trial, with the primary outcome incident or recurrent major depressive disorder.^12^ Short form 36 bodily pain score is based on 2 questions: “How much bodily pain have you had during the past 4 weeks?” and “During the past 4 weeks, how much did pain interfere with your normal work (including both work outside the home and housework)?” including 6 (none, very mild, mild, moderate, severe, very severe) and 5 (not at all, a little bit, moderately, quite a bit, extremely) response options, respectively. Short form 36 bodily pain was reported at baseline, posttreatment, and every 6 months over 36 months of follow-up. Short form 36 bodily pain scores range from 0 to 100, with higher scores representing lower pain intensity and less pain interference. In the United States, the population norm for SF-36 bodily pain is mean (SD) of 50.66 (16.28).^21^ In addition to changes in SF-36 bodily pain scores, we also defined another secondary outcome, minimal clinically important difference (MCID), as a 10-point increase at each follow-up relative to baseline; this threshold was previously validated.^2,39^

Given our hypothesis that sustained remission of insomnia would result in improvement in SF-36 bodily pain over 36 months of follow-up, we characterized remission of insomnia disorder by DSM-5 criteria after treatment (postintervention) and each of the follow-up assessments. Insomnia remission, sustained over the course of the follow-up, was defined by the absence of insomnia disorder at each assessment, from posttreatment to 36 month follow-up, or depression event per protocol for depression prevention trial.^12^

### 2.5. Sample size

The selective depression prevention randomized controlled trial was powered for the primary outcome, prevention of incident or recurrent DSM-5 major depression. For the present secondary analysis, the outcome was SF-36 bodily pain. A recent meta-analysis observed a pooled effect of CBT-I on bodily pain of d = 0.20.^36^ Although this is a small effect, the mixed models analysis of the current sample uses a highly reliable measure from the SF-36,^40^ yielding greater than 90% statistical power for detecting this effect size or larger.

### 2.6. Statistical analysis

The outcome, SF-36 bodily pain, was analyzed using linear mixed models to assess differences between the groups stratified by insomnia remission, given the hypothesis that sustained remission of insomnia would account for linear changes in bodily pain. The primary model controlled for baseline levels of SF-36, age, sex, and education with examination of linear trends for each group stratified by status of sustained remission of insomnia. These covariates were selected because prior studies have found that each of these variables contribute to the association between insomnia and self-reported pain outcomes.^17^ We tested whether data were missing at random and performed Little missing completely at random (MCAR) test; this result was χ^2^(115) = 107.15, *P* = 0.69. Given support for the assumption of MCAR, mixed linear models were used to test whether SF-36 bodily pain changed significantly over the follow-up in each of the 4 groups. Predefined analyses were also performed in which differences in SF-36 bodily pain were tested as a function of sustained insomnia remission status and as function of treatment group. Sensitivity analyses explored effects of missing data on the outcome bodily pain through an expectation-maximization estimation. Complete data sets were generated using an iterative Markov chain Monte Carlo method. For the MCID SF-36 bodily pain outcome, the mean value of the MCID response over follow-up was calculated, and difference as a function of sustained insomnia remission was tested using general linear modeling with a logistic link function. SPSS software version 28 (IBM Corp, Armonk, NY) was used for statistical analysis. A 2-sided *P* < 0.05 was considered statistically significant.

## 3. Results

### 3.1. Participants and treatments

As previously reported,^12^ 291 participants (mean [SD] age, 70.1 [6.7] years; 168 [57.7%] female; 7 [2.4%] Asian, 32 [11.0%] Black, 3 [1.0%] Pacific Islander, 241 [82.8%] White 6 [2.1%] multiracial, and 2 [0.7%] of unknown race and ethnicity) were enrolled, with 156 randomized to CBT-I and 135 to SET (Supplement, eResults, eFigure1, http://links.lww.com/PR9/A282). Baseline characteristics were previously described,^12^ and also found in Table 1. Baseline levels of SF-36 bodily pain did not differ between treatment groups (t_1,274_ = 0.31; *P* = 0.76) or between insomnia remission groups (t_1.274_ = 0.45; *P* = 0.90). Furthermore, there were no differences in SF-36 bodily when the sample was stratified by history of depression (t_1,274_ = 0.91, *P* = 0.37).

**Table 1:** Characteristics of participants by treatment group.*

Characteristic	CBT group (N = 156)	SET group (N = 135)
Age—y	70.2 ± 7.0	69.9 ± 6.4
Female sex—no. (%)	86 (55.1)	82 (60.7)
Race group—no. (%)†		
White	130 (83.3)	111 (82.2)
Black	16 (10.3)	16 (11.9)
Asian	3 (1.9)	4 (3.0)
Pacific Islander	3 (1.9)	0 (0)
Other	2 (1.3)	4 (3.0)
Unknown	2 (1.3)	0 (0)
Ethnicity—no. (%)		
Non-Hispanic/Non-Latino	142 (91.0)	130 (96.3)
Hispanic/Latino	12 (7.7)	5 (3.7)
Unknown	2 (1.3)	0 (0)
Marital status, no. (%)		
Married/partnered	72 (46.2)	64 (47.4)
Income‡	85.6 ± 49.7	78.7 ± 47.9
Employment, no. (%)		
Fulltime	49 (31.4)	49 (36.3)
Education—y	16.9 ± 2.7	16.4 ± 2.4
Body mass index§	27.1 ± 4.2	26.2 ± 4.3
Charlson Comorbidity Index‖	2.8 ± 1.0	2.8 ± 0.9
SF-36 bodily pain	67.9 ± 21.2	67.1 ± 22.1
Sleep disturbance¶		
*DSM-5* insomnia diagnosis—no. (%)	127 (81.4)	111 (82.2)
Duration of insomnia—mo	17.7 (25.5)	20.9 (27.2)
Athens insomnia score#	9.4 ± 3.4	9.5 ± 3.6
Use of hypnotic medications, no. (%)	33 (21.2)	24 (17.8)
Depression		
Depression history—no. (%)**	58 (37.2)	65 (48.1)
Use of antidepressants—no. (%)	25 (16.0)	20 (14.8)
PHQ-8 score††	3.4 ± 2.9	4.0 ± 3.1
History of other psychiatric comorbidity‡‡		
Generalized anxiety disorder—no. (%)	17 (10.9)	19 (14.1)
Alcohol use disorder—no. (%)	10 (6.4)	13 (9.6)

* Plus–minus values are means ± SD.

† Race and ethnicity was reported by the participants.

‡ Income is reported in 1000 dollars per year.

§ The body-mass index is the weight in kilograms divided by the square of the height in meters.

‖ The Charlson Comorbidity Index includes 17 categories of comorbidity, each with an assigned score of 1 to 6, depending on the risk of death associated with the condition; maximum score is 29.

¶ To be eligible for the study, all participants fulfilled *ICSD-2* and *DSM-IV* criteria for insomnia; number and percentage who also fulfilled *DSM-5* criteria for insomnia disorder are shown. After study onset, diagnostic criteria for *DSM-IV* insomnia disorder were revised by *DSM-5* to include criteria for frequency (sleep difficulties > 3 times per week) and duration (>3 months).

# The Athens Insomnia Score rates severity of sleep disturbance according to the *ICSD-2* for insomnia diagnosis. Scores range from 0 to 24 for the 8-item version, and a score of 6 or higher has optimal sensitivity and specificity for the diagnosis of insomnia.

** Lifetime history of depression is fulfilled by major depressive disorder in the *DSM-5* criteria, as determined following administration of the *Structured Clinical Interview*.

†† PHQ-9 scores each of the 9 criteria for major depressive disorder in the *DSM-5*, with as “0” (not at all) to “3” (nearly every day); maximum score of 27. All eligible participants had sleep disturbance; hence, PHQ-8 scored each of the criteria for major depressive disorder with the exception of insomnia, yielding a maximum score of 24. Scores below 5 on the PHQ-9 indicate none to minimal depression.

‡‡ Lifetime history of any anxiety disorder or substance use disorder using *DSM-5* criteria, as determined following administration of the *Structured Clinical Interview*. None of the participants fulfilled criteria for a current psychiatric disorder except for insomnia disorder. History of any other psychiatric disorder including eating disorder was identified in 11 participants (3.8%).

CBT, cognitive behavioral therapy; PHQ-8, patient health questionnaire-8; SET, sleep education therapy; SF-36, short form 36.

### 3.2. Adherence and follow-up retention

As previously reported, the treatment groups did not differ on measures of treatment expectancy, acceptability, adherence, attendance, or fidelity were similar.^12^ As compared with CBT-I, participants randomized to SET were more likely to complete the intervention (χ^2^ = 4.9, *P* = 0.03), although treatment adherence was high for both groups (CBT-I 89.7%, SET 96.3%). Rates of follow-up to 36 months were similar between the treatment groups (χ^2^ = 0.7, *P* = 0.41; Supplement, eResults, http://links.lww.com/PR9/A282).

### 3.3. Sustained insomnia remission

As previously reported, administration of CBT-I was more likely to produce a sustained remission of insomnia (41 [26.3%]) as compared with SET (26 [19.3%]; adjusted β = 0.56; 95% CI, 0.07–1.04; *P* = 0.03), although a substantial fraction of participants in both treatment groups achieved sustained remission of insomnia. Similar results were found using imputed data, with greater rates of sustained remission of insomnia disorder in CBT-I vs SET (adjusted β = 0.47; 95% CI, −0.11 to 1.05; *P* = 0.09).

### 3.4. Short form 36 bodily pain

**Figure 1.**
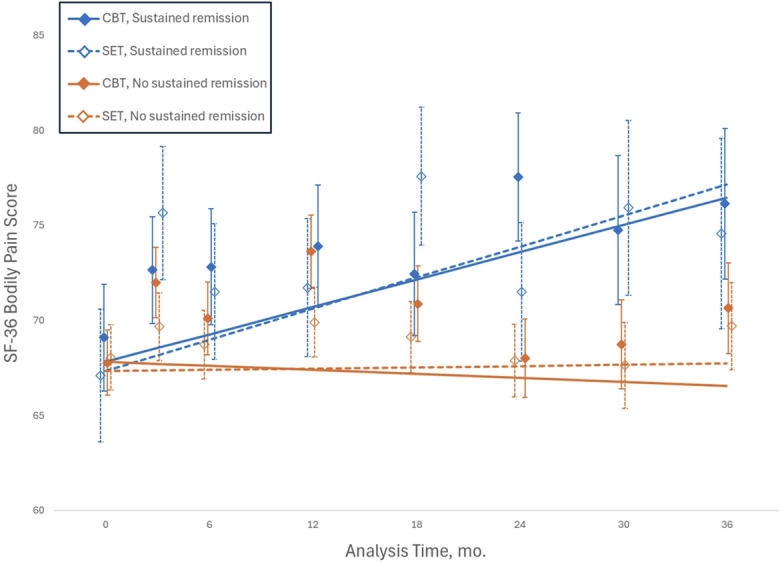
Change in SF-36 bodily pain by treatment group, stratified by insomnia remission status. Older adults without a chronic pain condition but with insomnia were randomized to receive cognitive behavioral therapy for insomnia (CBT-I) or sleep education therapy (SET). Sustained remission of insomnia was as defined by the absence of insomnia disorder at posttreatment and each follow-up assessment before a depression event, per protocol for depression prevention trial, or throughout 36 months of follow-up. SF-36 bodily pain with score showed increases or improvement over the duration of the study in CBT-I with insomnia remission (adjusted β *=* 0.18; 95% CI, 0.004–0.360; *P* = 0.045) and in SET with insomnia remission (adjusted β = 0.25; 95% CI, 0.035–0.457; *P* = 0.023) but not in CBT without insomnia remission (adjusted β *=* −0.048; 95% CI, −0.186 to 0.090; *P* = 0.50) or SET without insomnia remission (adjusted β *=* −0.02; 95% CI, −0.136 to 0.093; *P* = 0.72). SF-36, short form 36.

**Figure 2.**
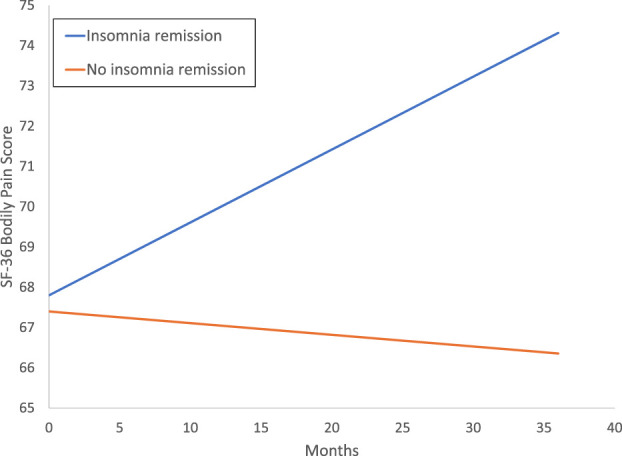
Change in SF-36 bodily pain by insomnia remission status. Older adults without a chronic pain condition but with insomnia were classified by insomnia treatment response following treatment with CBT-I or SET. Sustained remission of insomnia was as defined by the absence of insomnia disorder at posttreatment and each follow-up assessment before a depression event, per protocol for depression prevention trial, or throughout 36 months of follow-up. SF-36 bodily pain with score showed increases or improvement over the duration of the study in those classified as having insomnia remission (adjusted β = 0.19; 95% CI, 0.047–0.325; *P* = 0.009) not in those without insomnia remission (adjusted β *=* −0.03; 95% CI, −0.12 to 0.05; *P* = 0.51). CBT, cognitive behavioral therapy; SET, sleep education therapy; SF-36, short form 36.

To compare differences between the treatment groups stratified by sustained remission of insomnia (ie, CBT-I with and without sustained insomnia remission; SET with and without sustained insomnia remission), linear mixed models were used. Adjusting for baseline levels of SF-36 bodily pain, age, education, and sex, treatment groups stratified by sustained remission of insomnia did not differ (F_1,275.0_ = 0.02; *P* = 0.91). However, there was an overall time effect (F_7,1430.8_ = 1.98; *P* = 0.06) and a significant stratified treatment group by time interaction (*F*_3, 513.3_ = 3.8, *P* = 0.011). Linear models showed improvements in SF-36 bodily pain with score increases over the duration of the study in CBT-I with sustained insomnia remission (adjusted β *=* 0.18; 95% CI, 0.004–0.360; *P* = 0.045; d = 0.08) and in SET with sustained insomnia remission (adjusted β = 0.25; 95% CI, 0.035–0.457; *P* = 0.023; d = 0.17), but not in CBT without insomnia remission (adjusted β *=* −0.048; 95% CI, −0.186 to 0.090; *P* = 0.50; d = −0.02), or SET without insomnia remission (adjusted β *=* −0.02; 95% CI, −0.136 to 0.093; *P* = 0.72; d = 0.0; Fig. 1). Additional linear model analyses evaluated whether SF-36 bodily pain differed as a function of sustained insomnia remission in the total sample and found that sustained insomnia remission was associated with improvements in SF-36 bodily pain with score increases (adjusted β = 0.19; 95% CI, 0.047–0.325; *P* = 0.009) but not in those without insomnia remission (adjusted β *=* −0.03; 95% CI, −0.12 to 0.05; *P* = 0.51) (Fig. 2). Neither CBT-I nor SET showed significant changes in SF-36 bodily pain (*P's* > 0.8). Similar results were found using imputed data, as the pooled results from iterative Markov chain Monte Carlo method (n = 10) showed an identical pattern of significance and nonsignificance across the outcomes.

**Figure 3.**
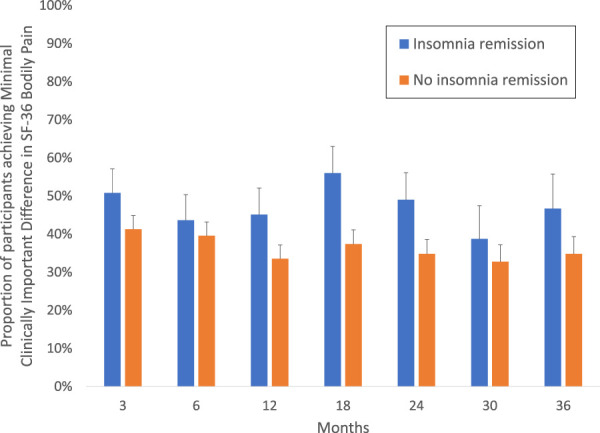
Difference in achieving a minimal clinically important response of SF-36 bodily pain by insomnia remission over follow-up. Proportion of older adults without a chronic pain condition but with insomnia were classified as achieving a minimal clinically important difference (MCID; increase of 10-points or more in SF-36 bodily pain) at each follow-up relative to baseline, by insomnia remission status. The likelihood of achieving a MCID response over the course of the trial was greater in those with insomnia remission (mean, 0.45; 95% CI, 0.39–0.52) as compared with the proportion in those without insomnia remission (mean, 0.35; 95% CI, 0.31–0.38; adjusted Likelihood ratio χ^2^_1, 16_ = 264.04; *P <* 0.001), with overall differences across all visits (F_1,403.5_ = 4.64; *P* = 0.03; Wald χ^2^(1) = 5.53, *P* = 0.02). SF-36, short form 36.

Additional analyses evaluated the impact of insomnia remission on bodily pain, as defined by proportion who showed a MCID response at each assessment. The proportion of participants who achieved MCID response over the course of the trial was greater in those with sustained remission of insomnia (mean, 0.45; 95% CI, 0.39–0.52) as compared with the proportion in those without insomnia remission (mean, 0.35; 95% CI, 0.31–0.38; adjusted Likelihood ratio χ^2^_1, 16_ = 264.04; *P <* 0.001), with overall differences across all visits (F_1,403.5_ = 4.64; *P* = 0.03; Wald χ^2^(1) = 5.53, *P* = 0.02; Fig. 3).

This study was conducted in community-dwelling older adults with insomnia and without chronic pain conditions, with a focus on depression prevention following insomnia treatment. Hence the trial was not designed to test whether the incident cases of chronic pain differed between the treatment groups. Nevertheless, we qualitatively evaluated this question. Using data obtained from the medical history, 6 participants developed new cases of chronic pain (CBT-I, N = 3; SET, N = 3). Furthermore, when groups were stratified by sustained insomnia remission or not, none of the cases of chronic pain occurred in those who had achieved sustained insomnia remission.

### 3.5. Monitoring of adverse events

No adverse events were observed during treatment. During follow-up, serious events were identified in the CBT-I group (4 illnesses and 1 death) and SET group (1 death); none were attributed to trial.

## 4. Discussion

In older adults with insomnia disorder, but without a chronic pain condition, sustained remission of insomnia after the administration of CBT-I or SET resulted in a significant improvement in bodily pain over 36 months of follow-up. By contrast, in the absence of remission of insomnia, scores of SF-36 bodily pain did not change. The overall benefit of insomnia remission on bodily pain was consistent in both treatment groups, as well as in the overall sample stratified by sustained insomnia remission. The level of improvement in bodily pain in response to insomnia remission is striking, given that this sample of older adults was relatively healthy with limited medical morbidity, was absent of comorbid chronic pain conditions, and only reported moderate pain complaints, which were less severe than the norm of the US older adult population.^21^ Finally, among those participants who achieved insomnia remission, a clinically important response was more likely to occur, with on average a 30% greater likelihood of achieving a clinically significant improvement in bodily pain, as compared with the proportion of those without insomnia remission. Together, these findings indicate that insomnia remission is key in improving moderate pain symptoms in community-dwelling older adults with insomnia without a chronic pain condition.

Pain is one of the most common complaints encountered by healthcare professionals who are treating adults older than 60 years. Yet, the prevalence of insomnia and pain symptoms in older adults stands in sharp contrast with the relative lack of randomized controlled trials conducted in older populations, which target insomnia to evaluate indirect benefits on pain.^32^ Given that moderate pain is prodromal to chronic pain conditions,^20^ this study is further significant; an improvement in moderate pain symptoms has implications for the prevention of chronic widespread pain or chronic pain conditions. For example, in the population-based HUNT study (n = 3105), moderate pain symptoms were associated with a nearly 2-fold greater risk of developing chronic widespread pain, which in turn is associated with declines in self-reported mental and physical health functioning,^20^ several fold increased risk of physical disability,^35^ and unfavorable prognosis at point of care.^22^ The benefit of insomnia treatment to reduce bodily pain might also translate into economic savings, as increasing severity of pain is linearly associated with increases in medical resource utilization in primary care patients,^18^ as well as in community-dwelling older adults.^4^ Given the lack of available pharmacological options and special vulnerabilities of older segments of our communities, targeting a modifiable risk factor (ie, insomnia) may be a particularly promising behavioral strategy to reduce pain and perhaps prevent chronic pain conditions. In addition, pain relief through nonpharmacologic insomnia treatment might reduce burden of polypharmacy in older adults, with positive effects on multiple health outcomes.^34^ Finally, in older adults with insomnia and without chronic pain conditions, this indirect approach to the treatment of bodily pain offers a new opportunity for the development of other innovative interventions to reduce bodily pain and possibly prevent the onset of a chronic pain condition. Specifically, older adults who simply report insomnia but do not have a chronic pain condition might be more readily engaged by insomnia treatment, as compared with CBT for pain. Furthermore, in these older adults with insomnia without a chronic pain condition, CBT-I might be viewed as less stigmatizing than pain management treatment strategies.^32^

This randomized controlled trial is one of the largest, with the longest follow-up, to answer an important clinical question about the effectiveness of insomnia treatment on moderate symptoms of bodily pain.^36^ Additional strengths include the internal validity of CBT-I vs SET, with evidence that these 2 treatments showed similar expectancy, acceptability, and adherence.^12^ We optimized dissemination potential by cost-effective delivery of treatments in a group format. In addition, given that telemedicine clinician delivery of CBT-I is noninferior to in-person CBT-I,^3^ dissemination could be readily expanded to remote communities. Also, as shown in our trial, well-designed SET could be a valid, scalable, less resource-intensive treatment option when CBT-I is unavailable, given that a substantial fraction of those who received SET also achieved sustained remission of insomnia. Moreover, effects were durable over 36 months, although patient burden was limited to only 16 hours of treatment exposure and no booster sessions.

Among the trials that have targeted insomnia in those with chronic pain conditions, almost none have examined the mechanisms that contribute to their effectiveness.^36^ We have found that insomnia is associated with inflammatory activation^11^ and that experimental sleep fragmentation causally induces hyperalgesia, which is mediated by the activation of cellular inflammation.^13^ Moreover, we and others have found that treatments such as CBT-I, which induce a remission of insomnia, also have the additional benefit of reducing systemic, as well as cellular and transcriptional markers of inflammation.^14–16^ Given that our primary selective depression prevention trial obtained measures of inflammation, further analyses can examine whether decreases in inflammation mediate improvements in bodily pain found in those older adults who achieved sustained insomnia remission.

### 4.1. Limitations

Women and those who are of races other than White have a disproportionate burden of risk of insomnia and pain^17^; hence, further research is needed to evaluate external validity to these subgroups. Second, dissemination of CBT-I because of limited access and cost of clinician delivered CBT-I.^26^ Although digitally delivered CBT-I may be more accessible and less costly, digital delivery of CBT-I shows lower rates of response and remission,^19^ greater attrition, and overall less durability.^5^ Given that the improvements in bodily pain are driven by sustained insomnia remission, it is possible that the benefits of CBT-I would be hampered by its digital delivery. Finally, the primary trial focused on the prevention of major depression in older adults with insomnia, and this secondary outcome analysis was limited to evaluation of temporal changes in pain intensity and pain interference, without further phenotyping of pain symptoms. The data captured by the pain-related SF-36 questions do not clearly indicate whether the pain represents development of new-onset chronic pain or acute pain throughout the past 4 weeks.

### 4.2. Conclusions

In this randomized clinical trial, sustained insomnia remission was associated with an improvement in bodily pain, as well as a greater likelihood of clinically meaningful improvement, in this community sample of older adults with insomnia. Given the high prevalence of sleep problems and moderate pain symptoms in older adults, and the robust relationship between insomnia and pain, treatments that effectively induce insomnia remission (eg, CBT-I) should be prioritized to reduce bodily pain with the potential to prevent the development of chronic pain conditions in older adults with insomnia.

## Disclosures

The authors have no conflict of interest to declare.

## Supplemental audio content

Supplemental digital content associated with this article can be found online at http://links.lww.com/PR9/A282.
